# Comparative studies on the histological characteristics of equine nasomaxillary aperture and paranasal sinus mucosa considering topographic and age-related differences

**DOI:** 10.1186/s13028-020-00534-2

**Published:** 2020-06-23

**Authors:** Alexander Schwieder, Christiane Pfarrer, Bernhard Ohnesorge, Carsten Staszyk, Astrid Bienert-Zeit

**Affiliations:** 1 Clinic for Horses, University of Veterinary Medicine Hannover, Buenteweg 9, 30559 Hannover, Germany; 2 Institute of Anatomy, University of Veterinary Medicine Hannover, Bischofsholer Damm 15, 30173 Hannover, Germany; 3 Institute for Veterinary-Anatomy, -Histology and -Embryology, Faculty of Veterinary Medicine of the Justus-Liebig-University Giessen, Frankfurter Str. 98, 35392 Giessen, Germany

**Keywords:** Horse, Nasomaxillary aperture, Histology, Sinusitis

## Abstract

**Background:**

Horses may acquire a range of paranasal sinus diseases. Clinical studies show slight differences regarding anatomical regions and age. Histopathological examination of tissue samples could play an important role in the diagnostic process. Therefore, detailed knowledge of the histological appearance of the paranasal sinus mucosa (PSM) and the nasomaxillary aperture mucosa (NAM) is essential. The objective of this study was to determine topographic and age-related differences within the healthy equine PSM. In addition, we aimed to gain detailed knowledge of the histological appearance of the NAM in comparison to the PSM.

**Results:**

The PSM had an average height of 75.72 ± 44.48 μm with a two-row pseudostratified columnar epithelium of 13.52 ± 4.78 μm. The parameters mucosal height, epithelial height and number of goblet cells revealed significant dependency of the sample site and age group. The maxillary and dorsal conchal sinus showed the highest values for these parameters. In terms of age, younger horses showed a significantly higher total mucosal height in contrast to a significantly lower epithelial height than older horses. Positive correlation was seen between the epithelial height and number of goblet cells. The NAM had an average height of 820.27 ± 653.21 μm. Its pseudostratified epithelium was usually arranged in three rows and had an average height of 44.9 ± 12.78 μm. The number of goblet cells in the NAM was five times higher than in the PSM. Serous glands were found in only 4% of the PSM samples and 100% of the NAM samples.

**Conclusions:**

There are significant histological differences between different paranasal sinus sites and between different groups of age. This may be related to an altered susceptibility for certain pathologies. The striking difference in the histological appearance of the NAM compared to the PSM could be due to an enhanced role in mucociliary clearance. Further studies are necessary to improve the understanding of mucosal function in specific paranasal sinus compartments and mucosal changes generated by different diseases.

## Background

The equine paranasal sinuses have been the focus of several studies in the recent past [1–9]. Different inflammatory, traumatic and neoplastic diseases are described including primary and secondary sinusitis, mainly of odontogenic origin, sinus cysts, progressive ethmoidal haematoma and neoplastic lesions in the paranasal sinuses [10–14].

The often chronic occurrence of these diseases, the complex anatomical structures within the equine skull and the difficulties of accessibility during diagnostic and surgical procedures impose specific challenges on veterinarians treating patients [11, 15]. Thus, paranasal sinus diseases are of great clinical relevance.

Pathologic conditions often affect more than one paranasal sinus compartment. The caudal maxillary and the rostral maxillary sinus are most often involved, followed by the ventral conchal and the conchofrontal sinus [16]. Slight differences in the distribution of some diseases are described. The rostral maxillary sinus, for example, is more often affected by primary sinusitis in comparison to sinus cysts [16]. The caudal maxillary sinus shows an increased occurrence of squamous cell carcinomas and bone tumours in comparison to other compartments [17].

The histopathological examination of mucosal samples could be an additional component in order to complete the clinical examination, diagnostic imaging and cytological and microbiological examination of secretions from the paranasal sinuses [18]. Tissue samples are of diagnostic value in the detection of neoplasia [17, 19, 20], progressive ethmoidal haematomas [11, 21] or necrosis of the nasal conchae [22].

Former histological descriptions of the respiratory mucosa within the equine paranasal sinuses are very limited [3, 18, 23–25], but of elementary importance.

Depending on the age, the paranasal sinuses undergo major anatomical changes, caused mainly by the expansion and eruption of the upper maxillary cheek teeth and the accompanying pneumatisation [26]. We hypothesize that there are differences in the histological appearance of the mucosal lining within different parts of the paranasal sinuses and different aged horses due to the complexity of the anatomical structures and their changes with increasing age and the distribution of individual diseases [16, 17].

Additionally, we hypothesize that the histological characteristics of the nasomaxillary aperture mucosa (NAM) represent a transitional zone between the mucosa of the nasal cavity and the paranasal sinuses and, therefore, the former differs histologically from the paranasal sinus mucosa (PSM).

The objective of this study was to gain more detailed knowledge of the histological appearance of the mucosa of the paranasal sinus and the nasomaxillary aperture (NA) in healthy horses. Furthermore, topographic and age-related differences in paranasal sinus histology were to be detected.

## Methods

### Horses

Seven equine skulls were used in a pretest of this descriptive, cross-sectional study to develop a suitable technique to gain and process equine PSM and NAM samples.

An additional 15 cadaveric horses, divided in three groups (group A: 0–5 years (y), group B: 6–15 y, group C: ≥ 16 y), were used to collect mucosal samples from different sites within the paranasal sinus system and from the NA. Each group consisted of five horses. All horses were euthanized or slaughtered for reasons not related to this study or to any disorders of the head. None of the horses had a known history or clinical signs of paranasal sinus disease.

### Sample collection

**Fig. 1:**
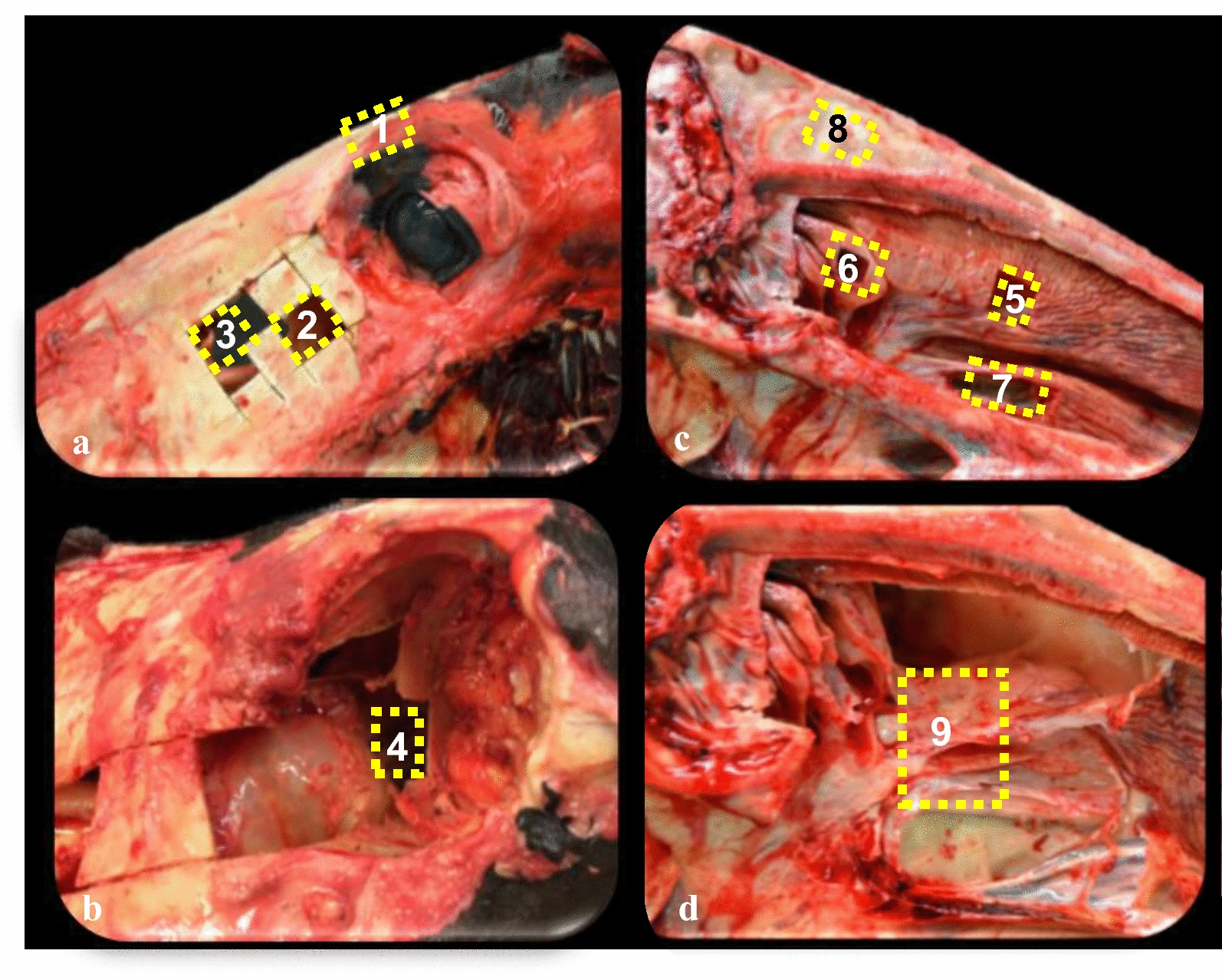
Photographs of a right-paramedian dissected equine skull (**a** + **b** left side, lateral views; **c** + **d** left side, medial view). During sample collection skin and musculature were transected (**a**), the eye and parts of the maxillary bone were removed (**b**), skulls were transected sagittally and the nasal septum was removed (**c**) and parts of the nasal conchae were removed (**d**). The yellow coloured boxes mark the nine different sample sites. **a** Frontal sinus, FS (1), caudal maxillary sinus, CMS (2), rostral maxillary sinus, RMS (3); **b** Caudal maxillary sinus, orbit, CMS-O (4); **c** Dorsal conchal sinus, DCS (5), middle conchal sinus, MCS (6), ventral conchal sinus, VCS (7) and frontal sinus septum, FSS (8); **d** Nasomaxillary aperture, NA (9)

All heads were separated from the neck within 10 min post-mortem. After removal of skin and musculature, the eyes were resected, and the skulls were transected transversally behind Triadan 03 and sagittally a few millimetres right-paramedian using a conventional band-saw. The left side of the skulls was used for all further investigations (Fig. 1). After resection of the nasal septum, samples with mucosa of the three nasal conchae, the NA, the maxillary and the frontal sinuses were taken. A total of eight different locations in the paranasal sinus compartments and one of the NA were used for sample collection. We aimed for a total of 135 different samples.

Different technical approaches to sample collection had failed during the pretesting period [29]. Finally, sampling mucosa in connection with the adjacent bone was successful. Sample collection from the dorsal (DCS), middle (MCS) and ventral conchal sinus (VCS) was performed using common surgical forceps and scalpel blades. Sample collection from the caudal maxillary sinus (CMS), caudal maxillary sinus, orbit (CMS-O), rostral maxillary sinus (RMS), frontal sinus (FS), frontal sinus septum (FSS) and NA was performed by using a motorised cutting wheel on a flexible shaft (Dremel^®^).

Subsequently, in samples which included thick adjacent bone (CMS, FS, RMS), this was thinned by using a motorised cylindrical burr on a flexible shaft (Dremel^®^) to reduce the time of chemical decalcification required. The flexible shaft was connected to a self-developed cooling system using saline solution to avoid overheating of the tissue samples.

### Histological procedure

Samples were placed in 10% formalin immediately after collection and fixed for 48 h. All samples underwent chemical decalcification (Ethylenediaminetetraacetic acid: EDTA, pH 7.0). The EDTA solution was renewed twice per week. Tissues were processed by routine methods, embedded in paraffin. Serial sections (3 µm) were cut and stained using Masson trichrome.

### Histological evaluation

A conventional light microscope (AXIO Scope.A1, Carl Zeiss Microscopy GmbH, Jena, Germany) connected to a high-resolution microscopic photo camera (Axiocam 105 color, Carl Zeiss Microscopy GmbH) was used for the histological evaluation. Sections were examined at different magnifications.

All samples were initially checked in terms of the integrity of the respiratory mucosa. Samples with mucosal or epithelial lining disruptions affecting at least two-thirds of the whole slice were excluded from further investigation.

All remaining samples were inspected to describe the histological characteristics of the respiratory mucosa. Microscopic images were taken subsequently in three different visual fields at 40× magnification. All images were reviewed blinded and the following histological parameters were evaluated using image analysis software (ZEN 2.3, Carl Zeiss Microscopy GmbH): mucosal and epithelial height; number of epithelial nuclei rows; shape of luminal epithelial cells; presence of mucus lining; presence, number and shape of goblet cells; alignment and density of cilia; presence of glands and vessels; presence and distribution of immune cells. All parameters were evaluated within a previously defined region of interest (ROI) of 37,500 μm^2^ (250 × 150 μm), which was positioned centrally within the sample. The mucosal and epithelial height were measured at two different locations within each ROI.

### Statistical analysis

Statistical analysis was performed using Statistics Software R (The R Foundation for Statistical Computing, Vienna, Austria). General linear models were fitted using the R package lme4 [27]. Two data types were analysed: metric data (epithelial height, mucosal height) and count data (number of goblet cells). Arithmetical mean values, standard deviations, medians and 25 and 75% quartiles of the individual examination parameters were calculated for the descriptive analysis.

The objective of the further statistical evaluation of all datasets was to detect differences between the individual age groups and the individual sample sites within the paranasal sinus system (CMS, CMS-O, DCS, FS, FSS, MCS, RMS and VCS). For all types of statistical analysis data of the NA were not considered. The metric datasets were checked for normal distribution using QQ plots. Subsequently, a generalized ANOVA was carried out, for which various general, mixed models were adapted to the data. The age groups and sample sites were assumed to be fixed factors and single animals and measurements as random factors. The data were then compared using the Chi-squared test to determine the significance of the fixed factors. The counting datasets displaying a Poisson distribution were analysed accordingly. Subsequently, the differences between age groups and sample sites were tested for significance for the individual examination parameters by means of the Tukey HSD (honestly significant differences) post hoc test. In addition, the Spearman’s test was used for correlation of the number of goblet cells with epithelial height.

The significance level was set to P ≤ 0.05 for all tests, with P-values rounded up, if necessary, in favour of the null hypothesis.

## Results

From 15 horses, 128 of 135 scheduled samples could be used for the histological investigation; 113 of these 128 samples originated from the PSM and the remaining 15 samples originated from the NAM. Three samples could not be collected because of the remaining long tooth roots within the maxillary sinuses and variances in the position of the maxillary sinus septum which interfered with the collecting process. Additionally, four samples did not reach the inclusion criteria due to an insufficient quality.

Details of the histological characteristics of the PSM and the NAM are listed in Table 1.

**Table 1:** Differences between histological features of paranasal sinus and nasomaxillary aperture mucosa

	PSM	NAM
Number of sites	8	1
Number of samples	113	15
Mucosal height (µm)	75.72 (± 44.48)	820.27 (± 653.21)
Epithelial height (µm)	13.52 (± 4.78)	44.9 (± 12.78)
Mucus lining (present in % of samples)	23% (26/113)	60% (9/15)
Cilia (alignment)	51% (58/113) aligned 49% (55/113) not aligned	40% (6/15) aligned 60% (9/15) not aligned
Cilia (density)	43% (49/113) high 54% (61/113) medium 3% (3/113) low	93% (14/15) high 7% (1/15) medium
Number of epithelial nuclei rows	2 (± 0)	3 (± 1)
Shape of luminal epithelial cells	77% (87/113) cubic 20% (23/113) columnar 3% (3/113) squamous	100% (15/15) columnar
Goblet cells (present in % of samples)	83% (94/113)	100% (15/15)
Goblet cells (number per ROI)	2 (± 3)	10 (± 8)
Goblet cells (shape)	100% (94/94) cup	93% (14/15) cup 7% (1/15) columnar
Vessels (present in % of samples)	100% (113/113)	100% (15/15)
Glands (present in % of samples)	4% (5/113)	100% (15/15)
Immune cells (present in % of samples)	88% (100/113)	93% (14/15)
Distribution of immune cells	92% (92/100) scattered 8% (8/100) diffuse	64% (9/14) scattered 29% (4/14) diffuse 7% (1/14) clustered

*NAM* Nasomaxillary aperture mucosa, *PSM* paranasal sinus mucosa, *ROI* region of interest

### Histological characteristics of the PSM

All samples consisted of respiratory mucosa adjacent to a bone plate of varying thickness. The bone plates in samples of the DCS, MCS, VCS and FSS were covered with respiratory mucosa on both sides (facing the paranasal sinuses and the nasal cavity); all other samples were covered with respiratory mucosa of the paranasal sinuses only. The PSM was significantly thinner than the NAM in all samples (see below).

**Fig. 2:**
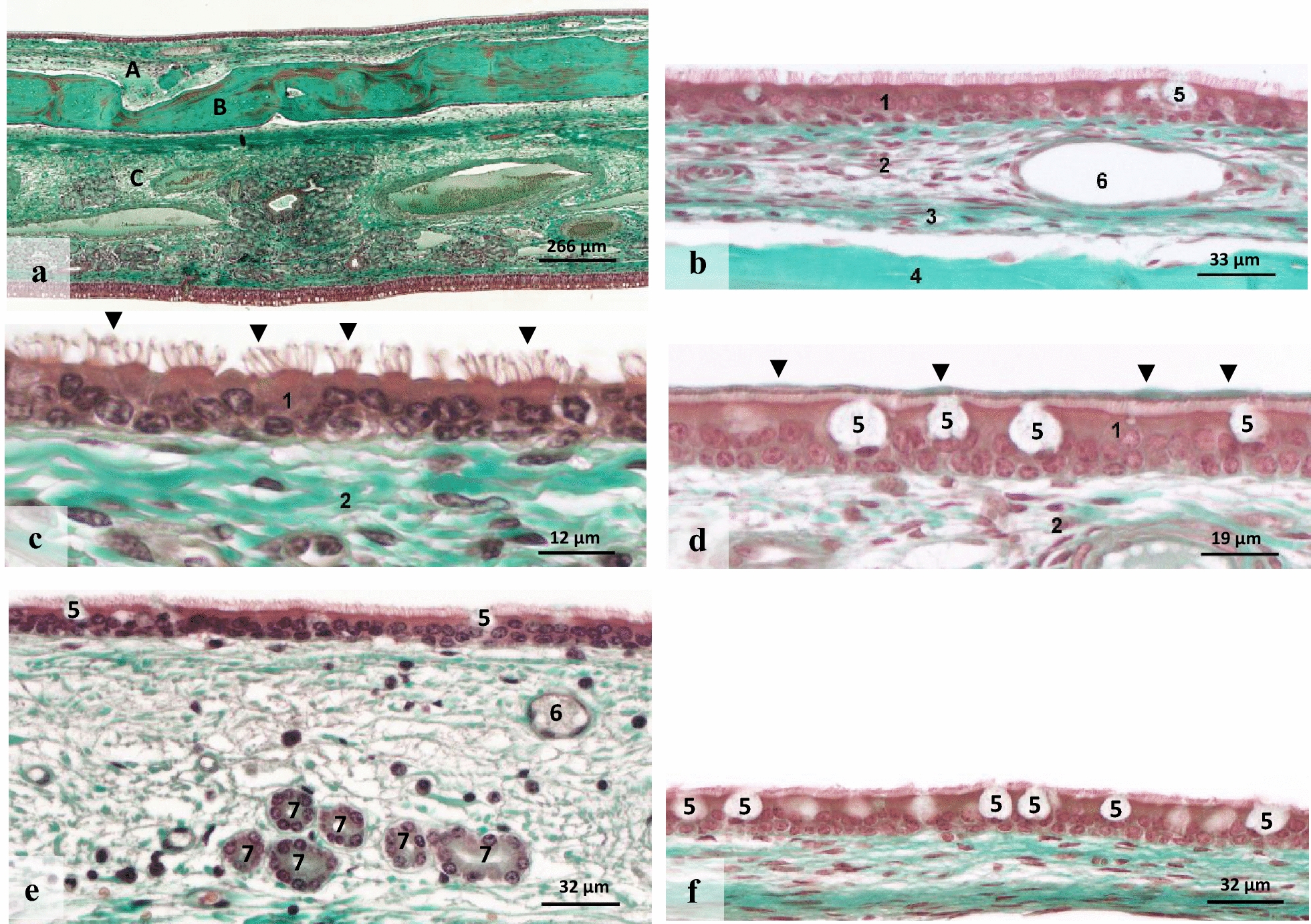
Histological characteristics and differences of the paranasal sinus mucosa in representative sample sites. **a** Overview illustrating all tissue layers of the DCS; **b** Overview illustrating the mucosa layers of the RMS; **c** Detail of epithelium displaying cilia on the surface (arrowheads) and several rows of nuclei [FS]; **d** Detail showing a thin mucus layer on the luminal surface of the ciliated pseudostratified columnar epithelium (arrowheads) with goblet cells [VCS]; **e**, **f** Age-related and topographical differences of the paranasal sinus mucosa (see Table 2): **e** Sample of the CMS from a 2 years old horse; **f** Sample of the MCS from a 12 years old horse. **f** A lower mucosal height, lower epithelial height and higher number of goblet cells compared to (**e**). A: Paranasal sinus mucosa; B: bone plate; C: nasal mucosa, 1: ciliated pseudostratified columnar epithelium; 2: lamina propria; 3: periosteum; 4: adjacent bone; 5: goblet cells; 6: blood vessel; 7: serous glands. *CMS* caudal maxillary sinus, *DCS* dorsal conchal sinus, *FS* frontal sinus, *MCS* middle conchal sinus, *RMS* rostral maxillary sinus, *VCS* ventral conchal sinus. Magnification see bars; Masson trichrome stain

Basic mucosal structures looked very similar in the various sinus compartments. A thin, ciliated pseudostratified columnar epithelium covered the subepithelial connective tissue, frequently traversed by vessels and rarely glands (Fig. 2).

The PSM had an average total height of 75.72 μm (± 44.48) throughout all the sinus compartments examined. The ciliated pseudostratified columnar epithelium had an average height of 13.52 μm (± 4.78) and displayed mostly two rows of epithelial nuclei. A thin lining with mucus on the apical surface of the ciliated epithelium was seen in 26 of 113 samples. Epithelial cells were cubic, columnar or squamous in shape and the epithelium was covered with fine cilia in every location. Subjectively evaluated, the density of cilia differed widely between the samples. Mucus-producing goblet cells interspersed with epithelial cells were found in 83% of samples. The number of goblet cells varied between 1 and 5 per ROI.

The subepithelial area consisted of loose connective tissue containing multiple vascular structures of different sizes. The entire vascular area within the ROI varied between 0 and 30,000 µm^2^. Glands existed only in 4% of these samples, all originating from the CMS or CMS-O. Lymphocytes and plasma cells were found in 100 of 113 samples, showing a scattered distribution pattern. Eight samples from different horses and sites showed a diffuse pattern with an accumulation of immune cells near vessels and glands.

### Histological characteristics of the NAM

**Fig. 3:**
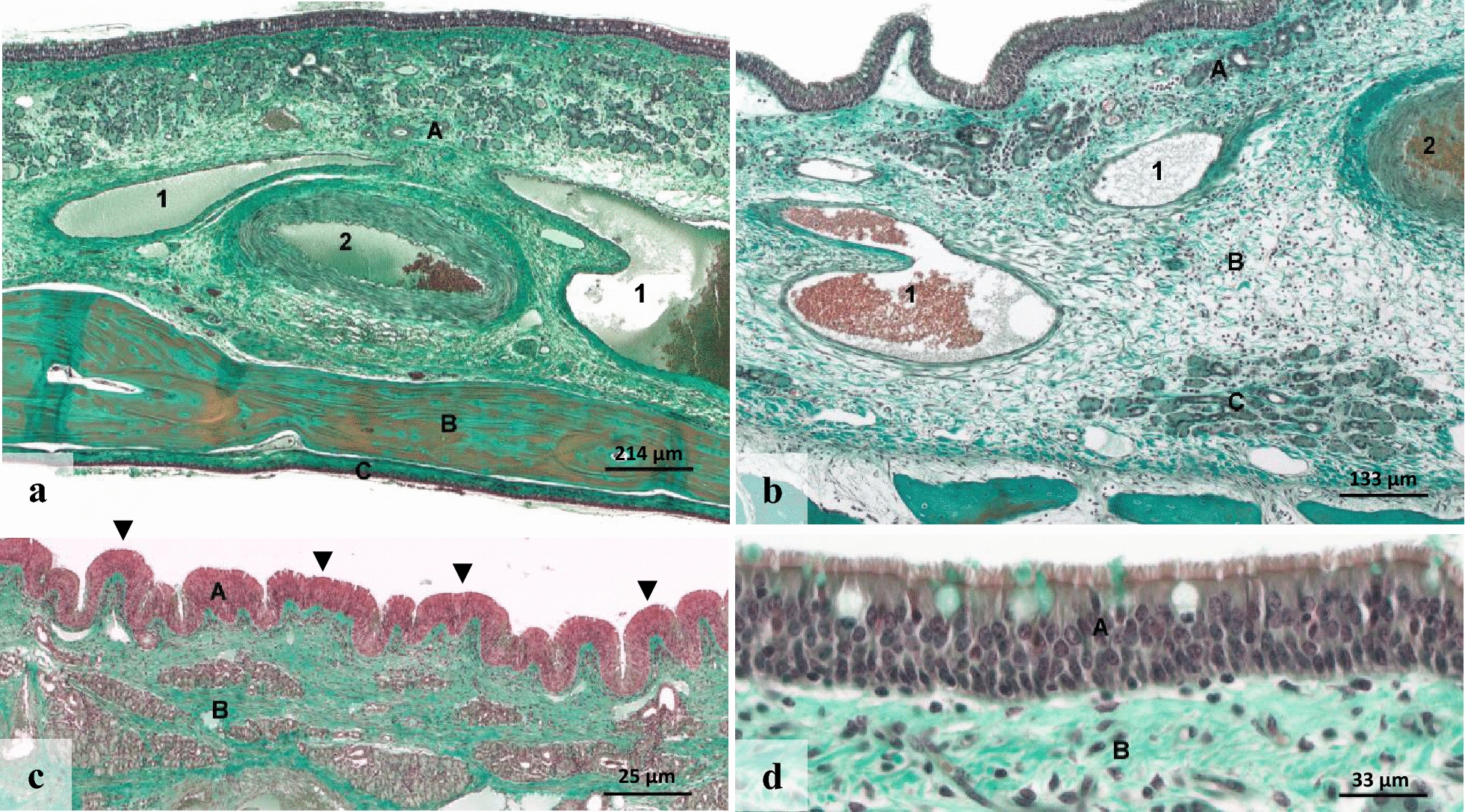
Histological characteristics of the nasomaxillary aperture mucosa. **a** Overview illustrating the nasomaxillary aperture mucosa (A) with large veins (1) and artery (2), adjacent bone (B) and paranasal sinus mucosa (C); **b** Overview of lamina propria (A–C) arranged in three zones. A, subepithelial top area, and C, bottom area, contain many serous glands, while B, middle area, contains large veins (1) and an artery (2); **c** The mucosa shows pronounced folding (arrowheads). A: epithelium, B: lamina propria; **d** Detail of high, ciliated pseudostratified columnar epithelium (A) with multiple rows of nuclei. B: Lamina propria; Magnification see bars; Masson trichrome stain

The histological characteristics of the NAM differed in specific parameters from the PSM (Fig. 3). The NAM had an average height of 820.27 μm (± 653.21). The mucosa frequently showed a pronounced folding. The samples were characterized by a typical, ciliated pseudostratified columnar epithelium with multiple rows of nuclei that covered the subepithelial tissue. This typical pseudostratified epithelium had an average height of 44.9 μm (± 12.78) with two to four rows of epithelial nuclei. The cilia regularly showed a high density. The epithelium in all samples contained mucus-producing goblet cells. Their number varied between 2 and 18. Mucus lining was found in 9 of 15 samples. The subepithelial lamina propria showed three distinct zones. In contrast to the PSM, very large veins and arteries dominated the central area. Their extent was not measured because it frequently exceeded the ROI of 37,500 µm^2^. Serous glands dominated the subepithelial area and the area next to the bony plate. Immune cells were found in 93% of NAM samples, distributed either sporadically, diffusely or in a clustered manner.

### Age-related and topographical differences of the PSM

Age-related and topographical differences are listed in Table 2. Datasets of mucosal height and epithelial height were distributed normally. The total mucosal height, epithelial height and number of goblet cells revealed a significant dependency of the age group tested (P < 0.05). Group A (≤ 5 y) showed a significantly higher mucosa compared to older horses (P < 0.001) (Fig. 2). By contrast, these young horses had a thinner epithelium (P < 0.001) and a lower number of goblet cells compared to older horses (P < 0.001).

**Table 2:** Detailed data of the different age groups and sample sites

	Mucosal height (μm)	Epithelial height (μm)	Goblet cells (number per ROI)
Group A (0–5 y)	103.18 (± 49.55)	12.16 (± 4.81)	1.3 (± 2.1)
Group B (6–15 y)	63.95 (± 35.85)	14.20 (± 4.82)	2.5 (± 3)
Group C (≥ 16 y)	61.72 (± 34.60)	14.11 (± 4.44)	2.3 (± 2.5)
CMS	120.60 (± 49.10)	17.75 (± 4.41)	2.9 (± 2.6)
CMS-O	88.32 (± 48.63)	14.52 (± 3.65)	3.5 (± 3.3)
DCS	92.47 (± 41.83)	16.14 (± 4.55)	3.0 (± 3.0)
FS	41.59 (± 17.78)	10.02 (± 3.97)	1.2 (± 1.7)
FSS	68.38 (± 39.07)	11.72 (± 2.64)	1.1 (± 1.5)
MCS	64.53 (± 28.04)	10.6 (± 3.98)	1.3 (± 2.4)
RMS	78.88 (± 37.38)	17.24 (± 4.19)	2.1 (± 2.0)
VCS	61.43 (± 42.30)	11.83 (± 3.50)	1.7 (± 2.6)
Total	75.72 (± 44.48)	13.52 (± 4.78)	2.1 (± 2.6)

*CMS* Caudal maxillary sinus, *CMS-O* caudal maxillary sinus, orbit, *FS* frontal sinus, *FSS* frontal sinus septum, *MCS* middle conchal sinus, *RMS* rostral maxillary sinus, *ROI* region of interest, *VCS* ventral conchal sinus, *y* years

Datasets of the mucosal height, epithelial height and number of goblet cells also revealed significant dependencies of the sample sites tested (P < 0.001). Samples of the CMS, CMS-O and DCS showed the highest mucosa among all samples, whereas samples of the FS showed the lowest mucosal height. The highest values for the epithelial height were measured at the CMS, CMS-O, DCS and RMS, whereas the lowest values were measured for the FS and MCS (Fig. 2). Goblet cells showed the highest numbers per ROI at the CMS, CMS-O and DCS. Lower numbers were counted at the FS, FSS and MCS. A positive correlation between the epithelial height and the number of goblet cells was seen (P < 0.001, r = 0.54).

## Discussion

The histological appearance of healthy respiratory mucosa within eight different sites of the equine paranasal sinuses and at the location of the NA were successfully evaluated by light microscopy.

Several pilot tests were performed to develop a suitable methodology [28]. In a first attempt, the collection of isolated respiratory mucosal samples stripped from the underlying bone through trephinations holes was performed. Most of these samples were severely damaged during handling due to the fragility of the healthy respiratory mucosa, making an appropriate histological examination impossible. However, mucosal thickening, which is a common finding in diseased horses [29, 30] could simplify the collection of isolated mucosal samples in diseased patients. Subsequently, mucosal samples were collected together with the adjacent bone. A motorised cutting wheel on a flexible shaft helped to cut the thick adjacent bone in different sites (CMS, CMS-O, NAM, RMS, FS and FSS). The bone plates were thinned down with a motorised cylindrical burr immediately after extraction to reduce their thickness and, thus, the time needed for EDTA decalcification. The EDTA-based ultrasound assisted decalcification [31] was also used in the pretesting period to reduce the time of decalcification but resulted in multiple soft tissue damage. As heat-associated soft tissue damage is a possible complication, for example, in equine dentistry when using rotating devices [32], cold saline solution was continuously applied to the bone surface during the mechanical processing. In the final histologic examination, four individual samples showed focal detachments of the mucosa from the underlying bone, which could have been caused by the mechanical processing or heat damage. Hence, these samples had to be excluded because of the predetermined inclusion criteria in matters of tissue integrity. No indicators of mechanical or heat-associated damage were observed in any other sample. All remaining samples were examined successfully.

The selection of sample sites aimed to gain a wide range of histological information concerning the entire paranasal sinus system and the NA. Moreover, sample sites chosen for the CMS, FS and RMS were correspondent to common surgical sites [13]; transferring the results obtained into clinical patients could be helpful. The NA is a slit-like duct between the nasal cavity and the paranasal sinus system and is thought to play a key role in the formation and healing of paranasal sinus diseases [30, 33–35].

The anatomical appearance and its fragility impeded sample collection of the NA. Thus, only one single location within this traditional zone has been chosen for this study.

Well-defined anatomical landmarks, such as the facial crest, the upper cheek teeth and the eye canthus, helped us to collect identically located samples in each skull. Computed tomography and associated three-dimensional reconstructions could have been used to determine the locations of sample collection even more precisely.

Only in young horses a few specific samples could not be taken at the predetermined locations of the maxillary sinuses, due to particular anatomical conditions. The variable position of the maxillary septum [36] and long tooth roots within the maxillary sinuses which were still present [34, 37] prevented collection of three samples. The sampling of younger horses during the pilot testing or prior computed tomography scans could have helped to avoid these challenges.

The study targeted the assessment of the histology of healthy paranasal sinus mucosa, although previous disease could not be completely precluded. In the present study, samples were taken immediately after death, to prevent respiratory mucosal samples from undergoing post-mortem changes, such as decay and autolysis.

According to the histological appearance of the respiratory mucosa, former literature describes a reduction of mucosal thickness between the nasal cavity and the paranasal sinuses in horses based on histological and radiologic assessments [3, 23, 35, 38]. The mucosal lining of healthy equine paranasal sinuses, for instance, was invisible in computed tomography images, due to its reduced thickness [35]. For the first time, the present study provides detailed information and objective measurements of histological features of the mucosal lining in the different equine paranasal sinuses and the NA. Older literature provides some limited details about the equine paranasal sinus mucosa and the NA [23, 24]. Measurements of the PSM in earlier literature varied between 50 and 200 µm [24]. The present study revealed a comparable mucosal thickness of 33 to 170 µm. The FS showed the lowest values among all sample sites. This is in accordance with a previous description [24], which delineated a progressive reduction in mucosal thickness along the different paranasal sinus compartments from the NA to the frontal or sphenopalatine sinus. Our results revealed that the mucosal height at the NA is about eleven times higher (820 µm) than the average height of the PSM. By contrast, earlier literature described a NAM height of 200 to 250 µm [24]. This large difference may be due to distinct changes in the mucosal thickness along the opening of the NA, which necessitates accurate sampling at exactly the same positions to get comparable results. To date, the mucosal lining at the NA has not been described in detail.

Furthermore, previous measurements of the inner mucosal lining of the nasal turbinates in young horses (1–2 y) [23] revealed two to three times higher values compared to the results of the present study in group A (≤ 5 y). Regarding these differences, the missing accordance in the selection of sample sites, different age distribution and differences in the techniques applied must be taken into account. Corresponding to previous descriptions [18, 23, 24], we found a pseudostratified columnar epithelium showing two rows of epithelial nuclei in the paranasal sinuses. Interestingly, the average epithelial height observed of 13.52 µm is in accordance with investigations in humans [39]. Our studies showed multiple significant differences between the paranasal sinuses regarding the different sample sites [27]. The highest epithelial measurements were seen in the dorsal conchal and maxillary sinuses, whereas the lowest epithelium was seen in the frontal sinus. Earlier investigations also stated that the highest epithelium was present within the caudal maxillary sinus and the lowest in the frontal sinus [24].

Concerning the NAM, the literature also provides evidence for a thickened epithelium in this site compared to the PSM [24], although detailed measurements are not available. Additionally, different authors previously reported a thicker epithelium within the nasal cavity [23, 40]. We measured the average epithelial height in the NAM, which is about three times higher than the epithelium of the PSM. This is in agreement with our hypothesis that the NAM represents a transitional zone between the mucosa of the nasal cavity and the PSM.

Regarding differences between the age groups, our data revealed a significantly larger total mucosal height in horses ≤ 5 y. Relating to this, hyperplasia of the pharyngeal soft tissue is a common finding in young horses. One study showed that 68 of 70 young racing Thoroughbreds showed evidence of pharyngeal hyperplasia or pharyngitis [41], this could also have an effect on the histological appearance of the PSM. In addition, PSM samples of young horses sometimes showed areas of incomplete ossified adjacent bone. The ongoing process of ossification could be marked by enhanced vascularization, which could lead to an enhanced mucosal thickness. Difficulties in defining a border between the respiratory mucosa and the incomplete ossified bone could also have evocated mismeasurements in these horses.

By contrast, our data revealed a reduced epithelial thickness in horses ≤ 5 y of age. As a possible explanation for this, the epithelium of young horses has been less frequently exposed to environmental material during their lifetime compared to older horses. Repeated exposition to dust could potentially result in an enhanced mucociliary clearance in older horses, which requires multiplication of mucus-producing goblet cells. In accordance with this hypothesis, horses older than 5 y of age showed a significantly higher number of goblet cells.

Moreover, the differences in the number of goblet cells observed in different sample locations could indicate disparities in the amount of mucus production in different paranasal sinus compartments. The DCS, CMS and CMS-O, for example, showed a higher number of goblet cells. Additionally, the CMS and CMS-O were the only two sample sites containing serous glands. The complex architecture of the equine paranasal sinus system and the associated difference in size of the communication pathways [35] could affect the potential of mucus production and the necessity of mucociliary clearance in different anatomical sites.

Mucosal swelling and, thus, obstruction of the slit-like NA can result in a lack of drainage from the entire paranasal sinus complex [34]. This may be related to the distinctly higher number of goblet cells in the NAM compared to the PSM observed in our results, and has been stated previously [24]. In addition, the density of cilia covering the luminal surface and the presence of mucus lining was much higher in NAM samples than in the PSM. However, the presence of mucus lining could be altered during the processing of samples. Scanning electron microscopic investigations [23, 25] and additional high speed imaging of mucosal samples, as performed in recent studies [42, 43], could be helpful to analyse the detailed morphology and beat pattern of the cilia lining in different sites and give us a better understanding of the cleaning process.

The subepithelial tissue in both the NAM and PSM consisted of loose connective tissue containing blood vessels and glands of varying sizes and numbers. Different authors describe an increased vascularization of the nasal mucosa compared to the paranasal sinuses mucosa in general [40, 44–46]. Large veins in the nasal cavity have a high capacity for blood accumulation, which can lead to a severe swelling of the nasal mucosa [45, 46]. Although the predetermined size of our ROI limited distinct measurements, the intense vascularization of the NAM observed illustrates the capability of swelling in this region, with the risk of a complete obstruction of the slit-like NA, as described previously [34]. Serous glands were only rarely seen in histological sections of the PSM, whereas they were a common finding at the NAM. A higher abundance of glands within the nasal cavity has also been described earlier [23, 47]. Measuring the concrete area of vessels and glands could provide detailed information about vascularization and mucus production. However, the orientation of these structures in correlation to the direction of section could have influenced the results.

The respiratory mucosa within the paranasal sinuses in humans plays an important role in the immune defence due to environmental antigens [48]. Different authors have stated a regular appearance of lymphocytes and plasma cells within the paranasal sinus mucosa in horses and men [23, 49–51], illustrating the immunologic potential of the so-called nasal-associated lymphoid tissue (NALT) [51]. According to this, we observed a diffuse distribution of these cells in most PSM and NAM samples, occasionally accumulating around vessels and glands. Specific immunohistochemical investigations are required for further investigations of the immunologic features within the equine paranasal sinus mucosa.

## Conclusions

The results of the present study indicate significant histological differences between individual paranasal sinus compartments and between horses of different age. As clinical healthy horses were used for this study, the results obtained reflect normal anatomic conditions. Further studies in horses with paranasal sinus disease are needed to evaluate the relation between histological characteristics and the susceptibility for certain pathologies.

The histological appearance of the NAM differs histologically in certain aspects from the mucosa of the paranasal sinuses. Its remarkable thickness in combination with numerous blood vessels might explain the predisposition for massive swelling causing closure of the NA.

The results obtained may reflect physiological differences in the respiratory mucosa of varying paranasal sinus compartments. Further studies are needed to investigate differences in the mucosal function and their clinical significance.

## Data Availability

The datasets used and analysed during the current study are available from the corresponding author on reasonable request.

## Abbreviations

CMS: Caudal maxillary sinus
CMS-O: Caudal maxillary sinus, orbit
DCS: Dorsal conchal sinus
FS: Frontal sinus
FSS: Frontal sinus septum
HSD: Honestly significant differences
MCS: Middle conchal sinus
NA: Nasomaxillary aperture
NAM: Nasomaxillary aperture mucosa
NALT: Nasal-associated lymphoid tissue
PSM: Paranasal sinus mucosa
RMS: Rostral maxillary sinus
VCS: Ventral conchal sinus
y: Years

## References

[1] Gergeleit H, Verspohl J, Rohde J, Rohn K, Ohnesorge B, Bienert-Zeit A (2018). A prospective study on the microbiological examination of secretions from the paranasal sinuses in horses in health and disease. Acta Vet Scand.

[2] Kaminsky J, Bienert-Zeit A, Hellige M, Rohn K, Ohnesorge B (2016). Comparison of image quality and in vivo appearance of the normal equine nasal cavities and paranasal sinuses in computed tomography and high field (3.0 T) magnetic resonance imaging. BMC Vet Res.

[3] Froydenlund T, Dixon P, Smith S, Reardon R (2015). Anatomical and histological study of the dorsal and ventral nasal conchal bullae in normal horses. Vet Rec.

[4] Arencibia A, Vázquez JM, Jaber R, Gil F, Ramiírez JA, Rivero M (2000). Magnetic resonance imaging and cross sectional anatomy of the normal equine sinuses and nasal passages. Vet Radiol Ultrasound.

[5] Fenner M, Verwilghen D, Townsend N, Simhofer H, Schwarzer J, Zani DD (2019). Paranasal sinus cysts in the horse: complications related to their presence and surgical treatment in 37 cases. Equine Vet J.

[6] Liuti T. Morphological assessment of paranasal sinuses and teeth in the horse. Ph.D. Thesis. Edingburgh: The University of Edinburgh; 2018. https://era.ed.ac.uk/handle/1842/33192. Accessed 05 Dec 2019.

[7] Liuti T, Reardon R, Smith S, Dixon P (2015). An anatomical study of the dorsal and ventral nasal conchal bullae in normal horses: computed tomographic anatomical and morphometric findings. Equine Vet J.

[8] Brinkschulte M, Bienert-Zeit A, Luepke M, Hellige M, Ohnesorge B, Staszyk C (2014). The sinonasal communication in the horse: examinations using computerized three-dimensional reformatted renderings of computed-tomography datasets. BMC Vet Res.

[9] Gergeleit H, Bienert-Zeit A, Seemann-Jensen A, Delarocque J, Ohnesorge B (2019). Cytological examination of secretions from the paranasal sinuses in horses. J Equine Vet Sci.

[10] Dixon PM, Parkin T, Collins N, Hawkes C, Townsend NB, Fisher G (2011). Historical and clinical features of 200 cases of equine sinus disease. Vet Rec.

[11] Tremaine WH, Dixon PM (2001). A long-term study of 277 cases of equine sinonasal disease. Part 1: details of horses, historical, clinical and ancillary diagnostic findings. Equine Vet J..

[12] Waguespack RW, Taintor J (2011). Paranasal sinus disease in horses. Compend Contin Educ Vet.

[13] Easley JT, Freeman DE (2013). New ways to diagnose and treat equine dental-related sinus disease. Vet Clin N Am Equine Pract.

[14] Dixon PM, Froydenlund T, Luiti T, Kane-Smyth J, Horbal A, Reardon RJM (2015). Empyema of the nasal conchal bulla as a cause of chronic unilateral nasal discharge in the horse: 10 cases (2013–2014). Equine Vet J.

[15] Beard W, Hardy J (2001). Diagnosis of conditions of the paranasal sinuses in the horse. Equine Vet Educ.

[16] Dixon PM, Parkin T, Collins N, Hawkes C, Townsend N, Tremaine WH (2012). Equine paranasal sinus disease: a long-term study of 200 cases (1997–2009): ancillary diagnostic findings and involvement of the various sinus compartments. Equine Vet J.

[17] Dixon PM, Head K (1999). Equine nasal and paranasal sinus tumours: part 2: a contribution of 28 case reports. Vet J.

[18] Tremaine WH, Clarke CJ, Dixon PM (1999). Histopathological findings in equine sinonasal disorders. Equine Vet J.

[19] Hilbert B, Little C, Klein K, Thomas J (1988). Tumours of the paranasal sinuses in 16 horses. Aust Vet J.

[20] Van Maanen C, Klein W, Dik K, Van den Ingh T (1996). Three cases of carcinoid in the equine nasal cavity and maxillary sinuses: histologic and immunohistochemical features. Vet Pathol.

[21] Barker W, Perkins J, Witte T (2013). Three horses with bilateral sinonasal progressive haematomas not associated with the ethmoidal labyrinth. Equine Vet Educ.

[22] Cehak A, Von Borstel M, Gehlen H, Feige K, Ohnesorge B (2008). Necrosis of the nasal conchae in 12 horses. Vet Rec.

[23] Kumar P, Timoney J, Southgate H, Sheoran A (2000). Light and scanning electron microscopic studies of the nasal turbinates of the horse. Anat Histol Embryol.

[24] Illig H. Über den histologischen Aufbau der Schleimhaut der Nebenhöhlen der Nase bei den Haussäugetieren. Dissertation. Stuttgart: Vereinige Medizinische Fakultät der Grossherzoglich Hessischen Ludwigs-Universität zu Giessen; 1910.

[25] Pirie M, Pirie H, Wright N (1990). A scanning electron microscopic study of the equine upper respiratory tract. Equine Vet J.

[26] Anatomie Staszyk C, Vogt C (2011). Lehrbuch der Zahnheilkunde beim Pferd.

[27] Bates D, Mächler M, Bolker B, Walker S. Fitting linear mixed-effects models using lme4; 2014. arXiv:14065823.

[28] Schwieder A. Untersuchung zur histologischen Beschaffenheit der Schleimhaut der Sinus paranasales des Pferdes unter Berücksichtigung von Topographie und Alter. Dissertation. Hannover: Tierärztliche Hochschule Hannover; 2018.

[29] Eggesbo HB (2006). Radiological imaging of inflammatory lesions in the nasal cavity and paranasal sinuses. Eur Radiol.

[30] O’Leary J, Dixon P (2011). A review of equine paranasal sinusitis. Aetiopathogenesis, clinical signs and ancillary diagnostic techniques. Equine Vet Educ.

[31] Guo X, Lam W-L, Leung K-S, Qin Y-X, Cheung W-H, Qin L (2008). Acceleration of bone decalcification by ultrasound. A practical manual for musculoskeletal research.

[32] Haeussler S, Luepke M, Seifert H, Staszyk C (2014). Intra-pulp temperature increase of equine cheek teeth during treatment with motorized grinding systems: influence of grinding head position and rotational speed. BMC Vet Res.

[33] Tatarniuk DM, Bell C, Carmalt JL (2010). A description of the relationship between the nasomaxillary aperture and the paranasal sinus system of horses. Vet J.

[34] Robinson EN, Furlow PW, McGorum BC, Dixon PM, Robinson EN, Schumacher J (2007). Anatomy of the respiratory system. Equine respiratory medicine and surgery.

[35] Probst A, Henninger W, Willmann M (2005). Communications of normal nasal and paranasal cavities in computed tomography of horses. Vet Radiol Ultrasound.

[36] Brinkschulte M, Bienert-Zeit A, Luepke M, Hellige M, Staszyk C, Ohnesorge B (2013). Using semi-automated segmentation of computed tomography datasets for three-dimensional visualization and volume measurements of equine paranasal sinuses. Vet Radiol Ultrasound.

[37] Dixon PM, Baker GJ, Easley J (2003). Anatomie der Zähne. Zahnheilkunde in der Pferdepraxis.

[38] Kaminsky J, Bienert-Zeit A, Hellige M, Ohnesorge B (2014). 3 tesla magnetic resonance imaging of the equine nasal cavities, paranasal sinuses and adjacent anatomical structures in 13 healthy horses. Pferdeheilkunde.

[39] Renovanz H-D, Nolte D, Renovanz H-D, Schumann K (1982). Die respiratorische Schleimhaut. Nase und Respirationstrakt - Obere und untere Luftwege als funktionelle Einheit.

[40] Liebich H-G, Liebich H-G (2010). Atmungsapparat. Funktionelle Histologie der Haussäugetiere und Vögel.

[41] Holcombe SJ, Robinson EN (2003). Medical treatment of upper airway dysfunction. Current therapy in equine medicine.

[42] Schipor I, Palmer JN, Cohen AS, Cohen NA (2006). Quantification of ciliary beat frequency in sinonasal epithelial cells using differential interference contrast microscopy and high-speed digital video imaging. Am J Rhinol.

[43] Ijaz F, Ikegami K (2019). Live cell imaging of dynamic behaviors of motile cilia and primary cilium. Microscopy.

[44] Reznik GK (1990). Comparative anatomy, physiology, and function of the upper respiratory tract. Environ Health Perspect.

[45] Michaels L, Hellquist HB, Michaels L, Hellquist HB (2012). The normal nose and paranasal sinuses. Ear, nose and throat histopathology.

[46] Dyce KM, Sack WO, Wensing CJG, Budras K-D, Goller H, Hofmann RR, Hummel G, Weyrauch KD (1991). Der Atmungsapparat. Anatomie der Haustiere.

[47] Rush B, Mair T, Rush B, Mair T (2004). Diseases of the nasal cavity and paranasal sinuses. Equine respiratory diseases.

[48] Reichel C (2019). Jüngste Entwicklungen in der Kopf-Hals-Immunologie. HNO.

[49] Ooi EH, Wormald P-J, Tan LW (2008). Innate immunity in the paranasal sinuses: a review of nasal host defenses. Am J Rhinol.

[50] Pronina OM, Koptev MM, Vynnyk NI, Proskurnya SA, Filenko BM (2018). Current view on the structure and function of the frontal sinus: literature review. Wiad Lek.

[51] Mair T, Batten E, Stokes C, Bourne F (1987). The histological features of the immune system of the equine respiratory tract. J Comp Pathol.

